# Linzagolix for Japanese women with uterine fibroids: a randomized, double-blind, placebo-controlled, phase 3 trial

**DOI:** 10.1016/j.xfre.2026.07.003

**Published:** 2026-07-21

**Authors:** Tasuku Harada, Yutaka Osuga, Hideomi Takahashi, Asuka Kawai, Fumiya Shimamura, KLH2302 Investigators, Masaki Hashimoto, Teruko Yasuda, Yukari Utsugisawa, Yuji Kashiwazaki, Rikako Uchida, Akiko Tanabe, Mariko Hatta, Sonoe Nishiguchi, Masuo Yoshioka, Akiyoshi Yamanaka, Masanori Maruyama, Hideki Hanashi, Jun Hayakawa, Ruriko Tsushima, Mamoru Urabe, Ryuji Nagai, Yuji Sutou, Shinya Iwasaki, Shohei Yoshida, Yukari Sumi

**Affiliations:** a Department of Obstetrics and Gynecology, Faculty of Medicine, Tottori University, Tottori, Japan; b Department of Obstetrics and Gynecology, Graduate School of Medicine, The University of Tokyo, Tokyo, Japan; c Clinical Research Department, Kissei Pharmaceutical Co., Ltd., Tokyo, Japan; d Clinical Projects Management Department, Kissei Pharmaceutical Co., Ltd., Tokyo, Japan

**Keywords:** Gonadotropin-releasing hormone receptor antagonist, heavy menstrual bleeding, linzagolix, pain, uterine fibroids

## Abstract

**Objective:**

To evaluate the efficacy, safety, and pharmacodynamics of linzagolix, an oral gonadotropin-releasing hormone (GnRH) receptor antagonist, in patients with heavy menstrual bleeding (HMB) and pain because of uterine fibroids.

**Design:**

Phase 3, multicenter, placebo-controlled, randomized, double-blind, parallel-group, confirmatory study (NCT05445167).

**Subjects:**

Premenopausal Japanese women with HMB and moderate-to-severe pain because of uterine fibroids.

**Intervention:**

Patients were randomized 1:1 to once-daily linzagolix 200 mg (n = 48) or placebo (n = 41) for 12 weeks.

**Main Outcome Measures:**

The primary endpoints were the proportion of patients with a total pictorial blood loss assessment chart (PBAC) score <10 from week 6–12, and the proportion of patients with a maximum numeric rating scale (NRS) score ≤1 during the 28 days before the end of treatment. Secondary endpoints included time to a total PBAC score <10 or maximum NRS score ≤1, analgesic use, blood hemoglobin, myoma and uterine volumes, patient-reported symptom severity and quality of life, and plasma estradiol levels. Safety endpoints included adverse events (AEs) and time to menstrual recovery.

**Results:**

The superiority of linzagolix vs. placebo was demonstrated for both the proportion of patients with a total PBAC score <10 from week 6–12 (91.5% vs. 2.5%; difference [95% confidence interval], 89.0% [76.0–96.2]), and the proportion of patients with a maximum NRS score ≤1 during the 28 days before the end of treatment (70.8% vs. 9.8%; difference, 61.1% [42.1–75.8]). Reductions in HMB and pain with linzagolix were rapidly achieved, occurred alongside improvements in other efficacy endpoints (e.g., analgesic use, blood hemoglobin, myoma and uterine volumes, patient-reported outcomes), and coincided with rapid and sustained reductions in plasma estradiol levels. The incidence of AEs was 91.7% in the linzagolix group and 75.6% with placebo; the most common AE in the linzagolix group was hot flush (47.9%). At the end of study treatment, mean plasma estradiol in the linzagolix group returned to pretreatment levels within 4 weeks, and the median time to menstrual recovery was 29 days.

**Conclusion:**

These data reaffirm that GnRH receptor inhibition with linzagolix is a tolerable and efficacious strategy to manage the signs and symptoms of uterine fibroids.

**Trial Registration:**

This trial was registered in clinicaltrials.gov, ID number NCT05445167. URL: https://clinicaltrials.gov/study/NCT05445167. Date of registration: 6/27/2022. Date of first enrollment: 10/11/2022.

Uterine fibroids are a common gynecological condition among women of reproductive age, characterized by benign tumors of the myometrium that develop in response to gonadal hormones (1). Although often asymptomatic and therefore undiagnosed, uterine fibroids are estimated to occur in >70% of women; at least 25% of them will present with clinically significant symptoms, commonly heavy menstrual bleeding (HMB) and associated anemia, abnormal uterine bleeding, dysmenorrhea, pelvic pressure, and pain (1, 2). These symptoms can profoundly impact several aspects of health-related quality of life (HRQoL), including productivity, sexuality, self-image, social functioning, mental health, and physical well-being (3, 4).

Gonadotropin-releasing hormone (GnRH) agonists (e.g., leuprorelin) are a common endocrine therapy for symptomatic uterine fibroids (5). By downregulating the hypothalamic-pituitary-gonadal axis and suppressing estrogen and progesterone production, GnRH agonists induce amenorrhea, reduce fibroid volume, and improve symptoms (6, 7, 8). However, GnRH agonists are also associated with an initial worsening of symptoms (the ‟flare-up effect”), caused by a transient increase in gonadotropin secretion, in addition to delayed pituitary recovery after the cessation of treatment (1, 5).

Linzagolix is an oral GnRH receptor antagonist in development for the treatment of symptomatic uterine fibroids and endometriosis (9, 10). In contrast to GnRH agonists, linzagolix directly inhibits pituitary GnRH receptors and gonadotropin secretion, and may therefore overcome the flare-up effect and delayed hormonal recovery seen with these existing therapies (5, 11). The elimination half-life of linzagolix is approximately 15 hours (12), which falls between that of other GnRH receptor antagonists (elagolix, 6 hours (13); relugolix, 25 hours (14)) and allows for once-daily dosing. On the basis of positive phase 3 trial data in Black and White populations (15, 16, 17), linzagolix was approved in Europe for the symptomatic treatment of uterine fibroids and endometriosis (12), but it is not currently approved in the United States. To support the regulatory approval of linzagolix in Japan, two recent phase 3 studies have sought to confirm its effects on fibroid-related symptoms in Japanese patients (KLH2301 [NCT05440383] and KLH2302 [NCT05445167]). Herein, we present the primary results from the KLH2302 study, which aimed to evaluate the efficacy, safety, and pharmacodynamic profiles of linzagolix vs. placebo in patients with HMB and pain caused by uterine fibroids in Japan.

## Materials and methods

### Study design

The KLH2302 study was a phase 3, multicenter, placebo-controlled, randomized, double-blind, parallel-group, confirmatory study of linzagolix in patients with HMB and pain because of uterine fibroids. The study was conducted at 21 sites in Japan from October 2022 to February 2024 (Supplemental Table 1, available online). The study was conducted in accordance with the Declaration of Helsinki, local regulations, and good clinical practice. The institutional review boards (IRBs) of participating sites approved the protocol before study commencement, and all patients provided written informed consent to participate. This study is registered with the Japanese Registry of Clinical Trials (jRCT2031220181), and the protocol and statistical analysis plan are available at ClinicalTrials.gov (NCT05445167).

### Study population

Eligible patients were premenopausal Japanese women aged ≥20 years, with uterine fibroids (≥1 surgically untreated and uncalcified myoma [subserosal, intramural, submucosal, and/or cervical] with a diameter ≥3 cm on transvaginal ultrasound) and a typical menstrual cycle (25–38 days, with ≥3 consecutive days of bleeding during menstruation). During the menstrual cycle immediately preceding the treatment period (i.e., the run-in period; described later), patients were also required to have HMB (defined as a total score ≥120 on the pictorial blood loss assessment chart [PBAC] (18)) and pain symptoms related to uterine fibroids (defined as a numeric rating scale [NRS] score ≥1 for ≥2 days, and a maximum NRS score ≥4).

Key exclusion criteria included a history of hematological diseases (including thalassemia, sickle cell anemia, folate deficiency, and coagulopathy; excluding iron deficiency anemia and latent iron deficiency); thyroid dysfunction and irregular menstruation (or suspected irregular menstruation because of thyroid dysfunction); lower abdominal pain because of irritable bowel syndrome or severe interstitial cystitis; pelvic inflammatory disease ≤8 weeks before screening; severe intermenstrual bleeding, significant anovulatory bleeding, or abnormal genital hemorrhage (on the basis of investigator judgment); and positive cervical cytology results ≤1 year before screening. Throughout the study, concomitant use of GnRH analogues or hormonal agents was prohibited, analgesics were permitted for severe pain related to uterine fibroids or adverse events (AEs), and oral iron supplements were permitted for patients already receiving iron before screening or for those with blood hemoglobin <10 g/dL at the start of the screening period. When analgesics were used to manage severe fibroid-related pain, investigators were instructed to keep the analgesic drug and dosage as consistent as possible.

### Study procedures

The study comprised a screening period, a single-blind run-in period, a double-blind treatment period of 12 weeks, and a follow-up period of up to 4 weeks (Supplemental Fig. 1, available online). All patients received placebo during the run-in period to minimize the placebo effect on pain symptoms. At the start of the treatment period (day 1–5 of the first menstruation during the run-in period), patients were randomized 1:1 to linzagolix 200 mg (two 100 mg tablets, once daily) or placebo (2 tablets, once daily) using an interactive web response system. Personnel who enrolled and assigned patients to study treatments did not have access to the random allocation sequence. The linzagolix dose selected for this study was in line with its approved posology in Europe (12), in which 200 mg once daily (providing full estradiol suppression (15)) is recommended for short-term treatment of <6 months.

Throughout the screening, run-in, and treatment periods, patients reported their symptoms using an electronic diary, in which menstrual bleeding (measured using the PBAC), pain (measured using the NRS), and analgesic use were recorded each day. Other assessments performed at prespecified study visits are presented in Supplemental Table 2.

### Study outcomes

Two primary endpoints were evaluated in this study: the proportion of patients with a total PBAC score <10 from week 6–12 of the treatment period; and the proportion of patients with a maximum NRS score ≤1 during the 28 days before the end of study treatment. Secondary endpoints reported herein included: patients with a total PBAC score <10 over time; time to a total PBAC score <10; patients with maximum NRS scores of ≤1 or 0 over time; maximum NRS score over time; blood hemoglobin, blood hematocrit, serum iron, and serum ferritin levels; myoma and uterine volumes (measured by transvaginal ultrasound); symptom severity, HRQoL total, and HRQoL subscale scores on the uterine fibroid symptom and quality of life (UFS-QOL) questionnaire (19); and patient-reported symptom change on the basis of the patient global impression of change (PGIC) scale. Post hoc efficacy endpoints included the time to a maximum NRS score ≤1 and the proportion of patients with analgesic use over time.

Prespecified pharmacodynamic endpoints were plasma estradiol and serum luteinizing hormone (LH), follicle-stimulating hormone (FSH), and progesterone levels. Safety endpoints included AEs, 12-lead electrocardiogram (ECG) parameters, clinical laboratory tests (hematology, blood biochemistry, and urinalysis), vital signs, body weight, and the time to menstrual recovery after the last dose of study treatment.

### Statistical analysis

Sample size calculations for the first primary endpoint assumed that 83% of the linzagolix group and 5% of the placebo group would achieve a total PBAC score <10 from week 6–12, on the basis of data from a previous trial of another GnRH receptor antagonist (20). For the other primary endpoint, sample size calculations assumed that 44% of the linzagolix group and 10% of the placebo group would achieve a maximum NRS score ≤1 in the 28 days before the end of treatment, on the basis of an exploratory analysis of data from the pivotal phase 3 PRIMROSE 1 and 2 trials (data on file) (15). Overall, a sample size of 78 patients (n = 39 per treatment group) was estimated to provide ≥90% power to detect the superiority of linzagolix vs. placebo for both primary endpoints, using Fisher’s exact tests with a two-sided significance level of 5%.

Efficacy and pharmacodynamic endpoints were assessed in the full analysis set, defined as all randomized patients who received ≥1 dose of study treatment. Safety outcomes were assessed in the safety set, defined as all patients who received ≥1 dose of study treatment during the treatment period. Unless otherwise stated, data were summarized using descriptive statistics and, where appropriate, with between-group differences and associated 95% confidence intervals (CIs).

For the two primary endpoints, the superiority of linzagolix over placebo was evaluated using Fisher’s exact tests. The primary objective of this study would only be achieved if the superiority of both primary endpoints was detected. Kaplan–Meier analyses and log-rank tests were used to evaluate the time to a total PBAC score <10, and the time to a maximum NRS score ≤1. Two-sample Wilcoxon tests were used to compare PGIC outcomes between groups. Statistical analyses were based on observed data with no imputation for missing values and were performed using SAS version 9.4 (SAS Institute Inc., Cary, NC).

## Results

### Study population

Among 148 patients who were screened for eligibility in this study, 89 patients with uterine fibroids and related HMB and pain were randomized to receive linzagolix (n = 48) or placebo (n = 41; Supplemental Fig. 2). Of these, 47 patients (97.9%) in the linzagolix group and 40 (97.6%) in the placebo group completed the 12-week treatment period. All 89 patients were included in both the full analysis and safety sets.

At baseline, the overall mean age was 43.0 years, and most patients (89.9%) had intramural uterine fibroids (Table 1). The mean total PBAC score at baseline was 301.4 (with a numerically lower mean score in the linzagolix group vs. placebo), and the mean maximum NRS score at baseline was 6.5 out of 10.

**Table 1:** Baseline patient demographics and clinical characteristics (full analysis set).

	Linzagolix (n = 48)	Placebo (n = 41)	Total (N = 89)
Age (y), mean (SD)	41.8 (5.4)	44.4 (6.0)	43.0 (5.8)
BMI (kg/m^2^), mean (SD)	23.6 (4.6)	24.4 (3.9)	24.0 (4.3)
Uterine fibroid type, n (%)^a^			
Subserosal	17 (35.4)	16 (39.0)	33 (37.1)
Intramural	42 (87.5)	38 (92.7)	80 (89.9)
Submucosal	4 (8.3)	2 (4.9)	6 (6.7)
Cervical	0	1 (2.4)	1 (1.1)
Comorbidities, n (%)			
Adenomyosis	7 (14.6)	5 (12.2)	12 (13.5)
Endometriosis	6 (12.5)	0	6 (6.7)
Total PBAC score, mean (SD)^b^	261.1 (154.1)	348.6 (235.7)	301.4 (199.7)
Maximum NRS score, mean (SD)^b^	6.8 (1.9)	6.2 (1.3)	6.5 (1.7)
Analgesic use, n (%)	35 (72.9)	26 (63.4)	61 (68.5)
Hemoglobin (g/dL), mean (SD)	11.9 (1.3)	12.3 (1.3)	12.1 (1.3)
<12 g/dL, n (%)	26 (54.2)	18 (43.9)	44 (49.4)
Myoma volume (cm^3^), mean (SD)	66.7 (73.3)	76.2 (156.4)	71.1 (118.4)
Uterine volume (cm^3^), mean (SD)	204.1 (122.1)	200.4 (200.3)	202.4 (161.9)
UFS-QOL symptom severity score, mean (SD)^c^	34.7 (16.3)	34.0 (18.2)	34.4 (17.1)
UFS-QOL HRQoL total score, mean (SD)^c^	68.7 (22.3)	71.2 (22.5)	69.8 (22.3)
Concern subscale	59.0 (30.0)	58.1 (29.9)	58.5 (29.8)
Activities subscale	73.7 (21.1)	76.4 (22.6)	75.0 (21.7)
Energy/mood subscale	64.9 (26.9)	68.6 (24.2)	66.6 (25.6)
Control subscale	72.8 (27.5)	75.2 (25.0)	73.9 (26.2)
Self-conscious subscale	75.4 (28.2)	77.9 (27.4)	76.5 (27.7)
Sexual function subscale	68.0 (31.6)	75.0 (34.0)	71.2 (32.7)

*Note:*^a^Patients with multiple types of uterine fibroids are counted in multiple rows.

b During the menstrual cycle immediately preceding the treatment period (i.e., run-in period).

c UFS-QOL symptom severity scores range from 0–100, with higher scores indicating greater symptom severity; UFS-QOL HRQoL total and subscale scores range from 0–100, with higher scores indicating greater HRQoL. BMI = body mass index; HRQoL = health-related quality of life; n = total sample size; N = total population size; NRS = numeric rating scale; PBAC = pictorial blood loss assessment chart; UFS-QOL = uterine fibroid symptom and quality of life.

### Efficacy outcomes

#### Menstrual bleeding and pain

For the first primary endpoint, 91.5% (95% CI: 79.6–97.6) of patients in the linzagolix group and 2.5% (95% CI: 0.1–13.2) in the placebo group achieved a total PBAC score <10 from week 6–12, yielding a between-group difference of 89.0% (95% CI: 76.0–96.2; Fig. 1A). Similarly for the other primary endpoint, 70.8% (95% CI: 55.9–83.0) of patients in the linzagolix group and 9.8% (95% CI: 2.7–23.1) in the placebo group achieved a maximum NRS score ≤1 in the 28 days before the end of treatment, yielding a between-group difference of 61.1% (95% CI: 42.1–75.8; Fig. 1B). The proportion of patients achieving each outcome was significantly greater in the linzagolix group vs. placebo (both *P*<.001), thereby establishing the superiority of linzagolix for both endpoints. Subgroup analyses of the coprimary endpoints in patients stratified by key baseline factors, including total PBAC score, which differed between treatment groups, yielded comparable results to the primary analyses and reaffirmed the efficacy of linzagolix (Supplemental Tables 3 and 4).

**Figure 1:**
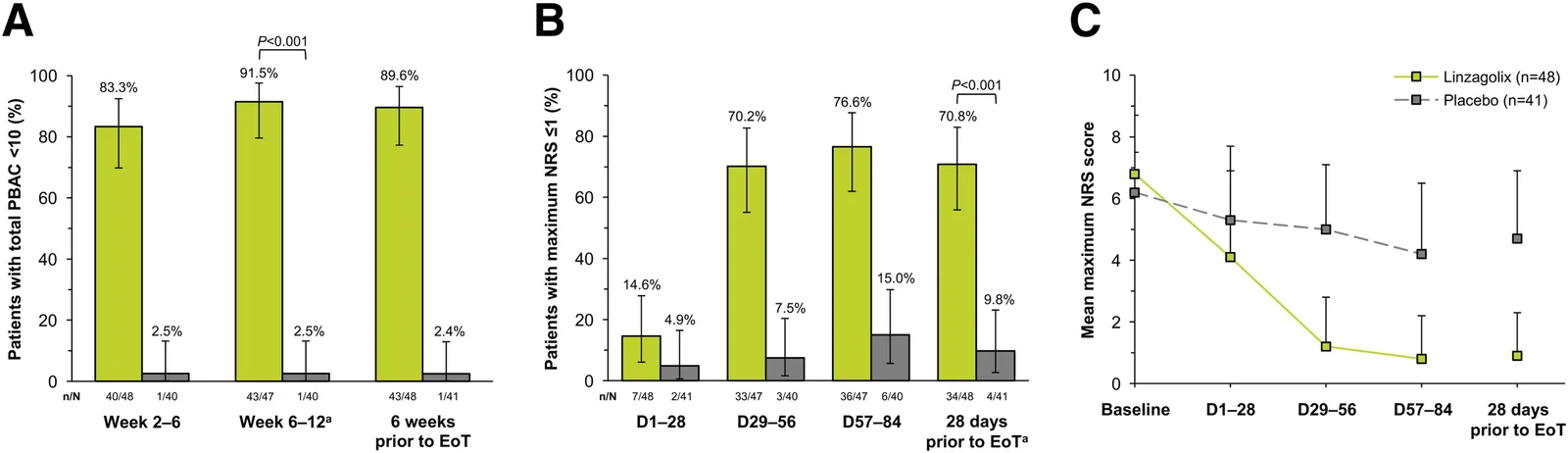
Proportion of patients with a total PBAC score <10 (**A**) or a maximum NRS score ≤1 (**B**) over time, and mean maximum NRS score over time (**C**). *Error bars* represent 95% CI in Fig. 1A and B, and standard deviation in Fig. 1C. ^a^Coprimary endpoint. The proportions of patients with a total PBAC score <10 from week 6–12 (Fig. 1A) and a maximum NRS score ≤1 in the 28 days before EoT (Fig. 1B) were significantly greater in the linzagolix group vs. placebo, on the basis of Fisher’s exact tests. CI = confidence interval; D = day; EoT = end of treatment; NRS = numeric rating scale; PBAC = pictorial blood loss assessment chart.

Regarding menstrual bleeding at other time points, significantly more patients in the linzagolix group vs. placebo achieved a total PBAC score <10 from week 2–6 (83.3% vs. 2.5%, respectively), as well as in the 6 weeks before the end of treatment (Fig. 1A). Accordingly, time to a total PBAC score <10 was significantly shorter in the linzagolix group vs. placebo (*P*<.001; Supplemental Fig. 3). The median time to a total PBAC score <10 was 5.0 days (95% CI: 3.0–6.0) in the linzagolix group, and not reached in the placebo group.

The proportion of patients with a maximum NRS score ≤1 was greater in the linzagolix group vs. placebo throughout the treatment period (Fig. 1B). Indeed, time to a maximum NRS score ≤1 was significantly shorter in the linzagolix group vs. placebo (*P*<.001; Supplemental Fig. 4). The median time to a maximum NRS score ≤1 was 13.5 days (95% CI: 5.0–42.0) in the linzagolix group, and not reached in the placebo group. The proportion of patients with a maximum NRS score of 0 was consistently higher in the linzagolix group vs. placebo throughout the treatment period (Table 2).

**Table 2:** Summary of secondary efficacy outcomes (full analysis set).

	Linzagolix (n = 48)	Placebo (n = 41)	Difference vs. placebo (95% CI)
Patients with maximum NRS score of 0, n/N (%)			
Day 1–28	6/48 (12.5)	1/41 (2.4)	10.1 (−1.9, 23.1)
Day 29–56	24/47 (51.1)	0/40 (0)	51.1 (36.1, 65.9)
Day 57–84	33/47 (70.2)	4/40 (10.0)	60.2 (40.9, 75.1)
28 d before EoT	32/48 (66.7)	2/41 (4.9)	61.8 (44.4, 75.8)
Patients with analgesic use, n/N (%)^a^			
Day 1–28	19/48 (39.6)	23/41 (56.1)	−16.5 (−36.8, 4.6)
Day 29–56	2/47 (4.3)	23/40 (57.5)	−53.2 (−69.1, −35.6)
Day 57–84	2/47 (4.3)	22/40 (55.0)	−50.7 (−66.8, −32.1)
28 d before EoT	3/48 (6.3)	25/41 (61.0)	−54.7 (−70.6, −36.6)
Hb at week 12 (g/dL), mean (SD)^b^	12.9 (1.0)	12.3 (1.5)	0.6 (0.1, 1.2)
Patients with Hb ≥12 g/dL at week 12, n/N (%)^b^^,^^c^	20/26 (76.9)	7/18 (38.9)	38.0 (8.1, 64.3)
Change in UFS-QOL symptom severity score from baseline at week 12, mean (SD)^d^	−27.9 (15.3)	−3.6 (17.1)	−24.3 (−31.2, −17.3)
Change in UFS-QOL HRQoL total score from baseline at week 12, mean (SD)^d^	19.9 (19.2)	3.7 (14.9)	16.3 (8.8, 23.7)
Concern subscale	33.6 (28.4)	5.6 (25.7)	28.0 (16.3, 39.6)
Activities subscale	18.5 (18.4)	2.5 (17.0)	16.0 (8.4, 23.6)
Energy/mood subscale	18.4 (24.0)	5.9 (14.4)	12.5 (3.9, 21.2)
Control subscale	16.0 (21.4)	3.9 (14.0)	12.1 (4.2, 20.0)
Self-conscious subscale	19.6 (27.7)	1.5 (22.5)	18.1 (7.2, 29.0)
Sexual function subscale	6.8 (38.6)	−2.2 (33.0)	9.0 (−6.6, 24.5)
PGIC at week 12, n (%)^b^^,^^e^	n = 46	n = 40	
Very much improved	16 (34.8)	0	34.8 (21.3, 50.2)
Much improved	16 (34.8)	3 (7.5)	27.3 (8.8, 43.9)
Minimally improved	9 (19.6)	12 (30.0)	−10.4 (−29.7, 8.3)
No change	4 (8.7)	25 (62.5)	−53.8 (−69.7, −33.8)
Minimally worse	0	0	–
Much worse	0	0	–
Very much worse	1 (2.2)	0	2.2 (−7.1, 11.7)

*Note:*^a^Post hoc endpoint.

b Between-group differences were evaluated in post hoc analyses.

c Analysis included patients with hemoglobin <12 g/dL at baseline.

d Reductions in UFS-QOL symptom severity scores from baseline indicate improvements in symptom severity; increases in UFS-QOL HRQoL total and subscale scores from baseline indicate improvements in HRQoL.

e Patients rated their overall symptoms because of uterine fibroids as ‟very much improved,” ‟much improved,” minimally improved,” ‟no change,” ‟minimally worse,” ‟much worse,” or ‟very much worse,” relative to their overall symptoms before the start of the run-in period. The distribution of patients across PGIC categories at week 12 was significantly different between treatment groups (*P*<.001, on the basis of two-sample Wilcoxon tests). CI = confidence interval; EoT = end of treatment; Hb = hemoglobin; HRQoL = health-related quality of life; n = total sample size; NRS = numeric rating scale; PBAC = pictorial blood loss assessment chart; PGIC = patient global impression of change; SD = standard deviation; UFS-QOL = uterine fibroid symptom and quality of life.

The mean maximum NRS score in the linzagolix group improved from 6.8 at baseline to 0.9 in the 28 days before the end of study treatment (Fig. 1C). Similarly, the proportion of linzagolix-treated patients who used analgesics for pain related to uterine fibroids decreased from 72.9% at baseline to 6.3% during the 28 days before the end of treatment, and was lower than the placebo group throughout the treatment period (Table 2).

#### Anemia

Treatment with linzagolix was associated with improvements in blood hemoglobin, blood hematocrit, serum iron, and serum ferritin levels over time (Table 2 and Supplemental Table 5). Among those with anemia (blood hemoglobin <12 g/dL) at baseline, 76.9% of patients in the linzagolix group achieved blood hemoglobin ≥12 g/dL at week 12, compared with 38.9% of those in the placebo group.

#### Myoma and uterine volumes

Greater reductions in myoma and uterine volumes were observed in the linzagolix group vs. placebo over the treatment period. At week 12, the mean percent change in myoma volume from baseline was −47.4% in the linzagolix group (vs. −1.0% in the placebo group); the corresponding percent change in uterine volume was −45.8% (vs. +6.4%).

#### Patient-reported outcomes

Linzagolix treatment was also associated with greater improvements in UFS-QOL scores and PGIC vs. placebo (Table 2). The UFS-QOL symptom severity scores, HRQoL total scores, and HRQoL subscale scores all improved in the linzagolix group from baseline at week 12. According to the PGIC, the proportion of patients whose uterine fibroid symptoms were “very much improved” or “much improved” at week 12 was 69.6% in the linzagolix group, vs. 7.5% in the placebo group. The distribution of patients across PGIC categories at week 12 was significantly improved in the linzagolix group vs. placebo (*P*<.001).

### Pharmacodynamic outcomes

In the linzagolix group, mean plasma estradiol was reduced to menopausal levels (<20 pg/mL) by week 4 of the treatment period, maintained through week 12, then returned to pretreatment levels by week 4 of the follow-up period (Supplemental Fig. 5). Similar trends were observed for serum LH, FSH, and progesterone levels (Supplemental Table 6). From week 4–12 of the treatment period, the proportion of patients with menopausal levels of plasma estradiol ranged between 91.7%–97.9% in the linzagolix group, vs. 10.3%–17.5% in the placebo group.

### Safety outcomes

The incidence of AEs was 91.7% in the linzagolix group and 75.6% in the placebo group, including treatment-related AEs in 81.3% and 41.5%, respectively (Table 3). Treatment-related AEs that occurred in ≥10% of patients in the linzagolix group were hot flush (47.9% vs. 0% in the placebo group), intermenstrual bleeding (41.7% vs. 31.7%), and hyperhidrosis (12.5% vs. 2.4%). Most AEs were mild in severity, and no severe or serious AEs were reported. One patient in the linzagolix group (2.1%) had an AE of abnormal hepatic function that led to treatment discontinuation; this patient had alkaline phosphatase levels that were above the upper limit of normal (ULN) at baseline and increased to <2-fold ULN at week 4, whereas alanine aminotransferase, aspartate aminotransferase, and bilirubin remained within normal reference ranges. This AE was mild in severity, deemed to be treatment-related by the investigator, and resolved after linzagolix discontinuation with no additional treatment required. Otherwise, no other AEs observed in this study resulted in treatment interruption or discontinuation.

**Table 3:** Summary of adverse events (safety set).

Patients with ≥1 AE, n (%)	Linzagolix (n = 48)	Placebo (n = 41)
Any AE	44 (91.7)	31 (75.6)
Mild	40 (83.3)	28 (68.3)
Moderate	4 (8.3)	3 (7.3)
Severe	0	0
Treatment-related AEs	39 (81.3)	17 (41.5)
AEs leading to treatment interruption	0	0
AEs leading to treatment discontinuation	1 (2.1)	0
Serious AEs	0	0
AEs leading to death	0	0
AEs occurring in ≥10% of patients		
Hot flush	23 (47.9)	0
Intermenstrual bleeding	20 (41.7)	13 (31.7)
Hyperhidrosis	6 (12.5)	1 (2.4)
COVID-19	5 (10.4)	2 (4.9)
Nasopharyngitis	4 (8.3)	5 (12.2)

*Note:* Adverse events (AEs) included events reported from the start of the treatment period to the end of the follow-up period, regardless of causality to study treatment. The AEs were coded using Medical Dictionary for Regulatory Activities thesaurus terms (MedDRA version 25.1). For any AEs by severity, patients with multiple AEs were counted only once, at the maximum severity. AE = adverse event; COVID-19 = coronavirus disease 2019; n = total sample size.

On 12-lead ECG, a marginal increase in the QT interval was observed after the first dose of study treatment in patients receiving linzagolix. No patient experienced a maximum QTcF interval increase of >60 ms from baseline, and no patient had a QTcF interval of >480 ms. Moreover, no clinically significant changes in laboratory parameters, vital signs, or body weight were observed.

After treatment, the average time to menstrual recovery was approximately 1 month among patients who received linzagolix. The median time from the last dose of study treatment to menstruation was 29 days (interquartile range, 28–32) in the linzagolix group and 17 days (8, 9, 10, 11, 12, 13, 14, 15, 16, 17, 18, 19, 20, 21, 22, 23) in the placebo group.

## Discussion

The results of this phase 3 study provide further evidence that linzagolix is a tolerable and efficacious treatment option for the signs and symptoms of uterine fibroids. In Japanese patients with HMB and pain because of uterine fibroids, linzagolix demonstrated superior reductions in menstrual bleeding vs. placebo from week 6–12 of the treatment period, as well as superior reductions in pain vs. placebo in the 28 days before the end of treatment. In addition to these primary endpoints, reductions in HMB and pain were rapidly achieved after linzagolix initiation, with a median time to onset of 5.0 days and 13.5 days, respectively. Similarly rapid improvements in menstrual bleeding were observed in the pivotal PRIMROSE 1 and 2 trials, where it was estimated that the median time to a clinically significant HMB reduction was 3 days with linzagolix 200 mg (16). In the present study, reductions in menstrual bleeding and pain were also maintained over 12 weeks of linzagolix treatment, and coincided with improvements in other efficacy outcomes (i.e., analgesic use, anemia-related laboratory parameters, myoma and uterine volumes, UFS-QOL scores, and PGIC). The efficacy of linzagolix was likely attributed to rapid and sustained reductions in circulating estradiol levels, without the initial flare-up effect seen with GnRH agonists. Linzagolix also demonstrated an acceptable safety profile overall, with no new safety signals detected. Overall, our findings were consistent with those of the PRIMROSE trials conducted in the United States and Europe (15, 16), and suggest that the efficacy and safety of linzagolix are maintained in Japanese women with uterine fibroids.

The results of this study were also consistent with a previous phase 3 trial of relugolix in Japanese women with uterine fibroids and HMB, which similarly used the proportion of patients with a total PBAC score <10 from week 6–12 as a primary efficacy endpoint (7). Although this endpoint for menstrual bleeding is more stringent than those used in other GnRH analogue trials (15), we suggest that this is appropriate given the full suppression dose of linzagolix 200 mg evaluated in this study. Data to inform the comparative efficacy of GnRH receptor antagonists for uterine fibroids are currently limited (21, 22); however, it should be noted that elagolix is administered twice daily, and the oral bioavailability of relugolix is affected by meals (13, 14). Given that linzagolix is administered once daily and its oral bioavailability is unaffected by meals, it may therefore represent a more convenient addition to this drug class. The rationale for evaluating a full suppression dose of linzagolix 200 mg in this study was that GnRH analogue treatment is currently limited to <6 months in Japanese clinical practice. However, a partial suppression dose of linzagolix 100 mg, with or without hormonal add-back therapy, is also approved for uterine fibroids in Europe (12), thereby allowing for individualized therapy and the potential for longer-term treatment.

Unlike other linzagolix trials, this study evaluated the efficacy of linzagolix for fibroid-related pelvic pain by enrolling patients with moderate-to-severe pain and assessing pain as a coprimary endpoint. We found that approximately 70% of patients in the linzagolix group had near-absent pain (NRS score ≤1) or no pain (NRS score of 0) at the end of treatment, and >90% did not require analgesics. Given that fibroid-related pain has a significant negative impact on HRQoL (23), marked reductions in pain with linzagolix may have also contributed to improvements in patient-reported outcomes. In particular, linzagolix improved UFS-QOL symptom severity and HRQoL scores to levels at week 12 that were equivalent to, or better than, women without uterine fibroids (19). Furthermore, PGIC data showed that approximately 70% of patients perceived linzagolix as effective (i.e., symptoms were “very much improved” or “much improved” at week 12). These findings underscore the potential utility of linzagolix for the management of pain caused by uterine fibroids.

Similar to previous studies of linzagolix and other GnRH receptor antagonists (7, 15, 24, 25), this study showed that reductions in myoma and uterine volumes occurred alongside improvements in menstrual bleeding and pain. Fibroid growth and uterine enlargement are associated with symptoms including pelvic pressure, frequent urination, and constipation (1); therefore, reducing myoma and uterine volumes with linzagolix may alleviate these symptoms, and in patients indicated for surgery, may also facilitate less-invasive procedures (e.g., laparoscopic myomectomy).

Although the efficacy of linzagolix and other GnRH analogues is attributed to their inhibitory effects on gonadotropin secretion, the hypoestrogenic state induced by these agents is also associated with menopause-like AEs (5). Similar to the PRIMROSE trials (15), the most common AE observed among linzagolix-treated patients in this study was hot flush; however, all hot flush AEs were mild or moderate in severity, and none warranted treatment discontinuation. Moreover, circulating estradiol levels were promptly restored after treatment and led to menstrual recovery after an average of 1 month, suggesting that GnRH receptor inhibition with linzagolix may overcome the delayed pituitary recovery associated with GnRH agonists (5).

Limitations of this study are related to its location, design, and duration. First, this study was conducted in Japan, which may limit its generalizability; however, comparable results in the PRIMROSE trials (15, 16) indicate that linzagolix is well tolerated and efficacious across a diverse range of patients. Second, because this study was a placebo-controlled trial conducted over 12 weeks of treatment, it does not inform the comparative efficacy of linzagolix vs. other GnRH analogues, nor longer-term treatment outcomes. Given the short duration of study treatment and follow-up, bone mineral density was not assessed in this study; however, we acknowledge that loss of bone mineral density is a common AE of longer-term GnRH analogue treatment (1, 5, 15). These limitations will be partly addressed by the phase 3 KLH2301 study conducted in parallel (NCT05440383), which aimed to compare the 24-week efficacy, safety, and pharmacodynamics of linzagolix vs. leuprorelin in patients with uterine fibroids and HMB.

## Conclusion

This phase 3 study evaluated the efficacy, safety, and pharmacodynamics of linzagolix in patients with HMB and pain associated with uterine fibroids. Reductions in menstrual bleeding and pain with linzagolix occurred alongside improvements in anemia, myoma and uterine volumes, patient-reported symptom severity, and HRQoL; and coincided with rapid and sustained reductions in estradiol levels over 12 weeks of treatment. Overall, these data suggest that GnRH receptor inhibition with linzagolix represents a tolerable and efficacious treatment strategy for patients with symptomatic uterine fibroids.

## Declaration of Interests

T.H. reports medical writing and clinical trial advisory fee payment was made to me based on medical expert contract with Kissei Pharmaceutical Co., Ltd. for the submitted work. Y.O. reports medical writing and clinical trial advisory fee payment was made to me based on medical expert contract with Kissei Pharmaceutical Co., Ltd. for the submitted work. H.T. is an employee of Kissei Pharmaceutical Co., Ltd.and holds patents issued to the company. A.K. is an employee of Kissei Pharmaceutical Co., Ltd. F.S. is an employee of Kissei Pharmaceutical Co., Ltd.
