# Supplementary material for: Linzagolix for Japanese women with uterine fibroids: a randomized, double-blind, placebo-controlled, phase 3 trial
Source: F S Rep. 2026 Jul 21;7(5):417–24. doi: 10.1016/j.xfre.2026.07.003 (PMC13681271; doi:10.1016/j.xfre.2026.07.003)
Supplement: Supplementary file 1 [file mmc1.docx]

# Supplemental Data

**Title:** Linzagolix for Japanese women with uterine fibroids: a randomized, double-blind, placebo-controlled, phase 3 trial

**Authors:** Tasuku Harada, M.D., Ph.D., Yutaka Osuga, M.D., Ph.D., Hideomi Takahashi, M.Ag., Asuka Kawai, B.S., Fumiya Shimamura, M.Eng., and KLH2302 Investigators

**Corresponding Author:** Asuka Kawai, B.S., Clinical Projects Management Department, Kissei Pharmaceutical Co., Ltd., 3-1-3 Koishikawa, Bunkyo, Tokyo, Japan. Phone: +81-(0)70-2238-3110; Email: [asuka_kawai@pharm.kissei.co.jp](mailto:asuka_kawai@pharm.kissei.co.jp)

## Supplemental Table 1

KLH2302 study investigators

| **Investigator name^a^** | **Study site** |
| --- | --- |
| Masaki Hashimoto | Hashimoto Clinic, Hokkaido |
| Teruko Yasuda | Yoshio Clinic, Hokkaido |
| Yukari Utsugisawa | Women's Clinic LUNA Yokohama Motomachi, Kanagawa |
| Yuji Kashiwazaki | KASHIWAZAKI OB/GYN CLINIC, Saitama |
| Rikako Uchida | Rikako Ladies Clinic, Osaka |
| Akiko Tanabe | jMOG Medical Corporation Tanabe Ladies' Clinic, Osaka |
| Mariko Hatta | Iryohojinshadan Seijunkai Juno Vesta Clinic Hatta, Chiba |
| Sonoe Nishiguchi | Asahi-Clinic, Kagawa |
| Masuo Yoshioka | SUZURAN CLINIC, Tokyo |
| Akiyoshi Yamanaka | Chayamachi Ladies Clinic, Osaka |
| Masanori Maruyama | Maruyama Memorial General Hospital, Saitama |
| Hideki Hanashi | New Medical Research System Clinic, Tokyo |
| Jun Hayakawa | Hayakawa Clinic, Osaka |
| Ruriko Tsushima | Tsushima Ruriko Women's Life Clinic Ginza, Tokyo |
| Mamoru Urabe | Omi Medical Center, Shiga |
| Ryuji Nagai | Kumamoto Shinto General Hospital, Kumamoto |
| Yuji Sutou | Sutou Ladies Clinic, Osaka |
| Shinya Iwasaki | Shizuoka City Shimizu Hospital, Shizuoka |
| Shohei Yoshida | Ginza Yoshida Clinic, Tokyo |
| Yukari Sumi | TORANOMON WOMENS CLINIC, Tokyo |

^a^One investigator did not give consent to be named in this publication.

## Supplemental Table 2

Study assessments

|  | **Screening period^a^** | **Run-in period^a^** | **Treatment period** | | | | **Follow-up period^b^** |
| --- | --- | --- | --- | --- | --- | --- | --- |
|  |  |  | **Baseline** | **Week 4** | **Week 8** | **Week 12** | **Week 16** |
| Visit | 1 | 2 | 3 | 4 | 5 | 6 | 7 |
| Patient characteristics | X | X | X |  |  |  |  |
| Symptom diary (PBAC, NRS, analgesic use) | → | → | → | → | → | → |  |
| Clinical laboratory tests^c^ | X | X | X | X | X | X | X |
| Myoma and uterine volumes^d^ | X |  | X | X | X | X |  |
| UFS-QOL |  |  | X |  |  | X |  |
| PGIC |  |  |  |  |  | X |  |
| Pharmacodynamic tests^e^ |  |  | X | X | X | X | X |
| Adverse events | → | → | → | → | → | → | → |
| Vital signs | X | X | X | X | X | X | X |
| Body weight | X | X | X | X | X | X | X |
| 12-lead electrocardiogram | X | X | X | X | X | X | X |
| Menstrual recovery |  |  |  |  |  |  | X |

NRS = numeric rating scale; PBAC = Pictorial Blood Loss Assessment Chart; PGIC = Patient Global Impression of Change; UFS-QOL = Uterine Fibroid Symptom and Quality of Life.

^a^The screening period was defined as the time between the screening visit and the next visit during day 1–5 of the first menstruation after screening (visit 2); the run-in period was defined as the time between visit 2 and the next visit during day 1–5 of the second menstruation after screening (visit 3).

^b^For patients who discontinued study treatment before week 12, the follow-up period was 4 weeks after the last dose.

^c^Clinical laboratory tests included hematology, blood biochemistry, and urinalysis.

^d^Myoma and uterine volumes were measured by transvaginal ultrasound.

^e^Pharmacodynamic tests included plasma estradiol, serum luteinizing hormone, serum follicle-stimulating hormone, and serum progesterone.

## Supplemental Table 3

Proportion of patients with a total PBAC score <10 from week 6–12 in subgroups stratified by baseline factors (full analysis set)

| **n/N (%) (95% CI)** | **Linzagolix (n=48)** | **Placebo (n=41)** |
| --- | --- | --- |
| Age (years) |  |  |
| <40 | 13/14 (92.9) (66.1, 99.8) | 0/5 (0) (0.0, 52.2) |
| ≥40 | 30/33 (90.9) (75.7, 98.1) | 1/35 (2.9) (0.1, 14.9) |
| BMI (kg/m^2^) |  |  |
| <25 | 31/35 (88.6) (73.3, 96.8) | 0/23 (0) (0.0, 14.8) |
| ≥25 | 12/12 (100) (73.5, 100.0) | 1/17 (5.9) (0.1, 28.7) |
| Total PBAC score |  |  |
| <200 | 19/20 (95.0) (75.1, 99.9) | 0/12 (0) (0.0, 26.5) |
| ≥200 | 24/27 (88.9) (70.8, 97.6) | 1/28 (3.6) (0.1, 18.3) |
| Comorbid endometriosis^a^ |  |  |
| No | 39/41 (95.1) (83.5, 99.4) | 1/40 (2.5) (0.1, 13.2) |
| Yes | 4/6 (66.7) (22.3, 95.7) | – |
| Birth experience |  |  |
| No | 19/22 (86.4) (65.1, 97.1) | 0/19 (0) (0.0, 17.6) |
| Yes | 24/25 (96.0) (79.6, 99.9) | 1/21 (4.8) (0.1, 23.8) |
| Uterine volume (cm^3^) |  |  |
| ≤170 | 26/27 (96.3) (81.0, 99.9) | 0/22 (0) (0.0, 15.4) |
| >170 | 17/20 (85.0) (62.1, 96.8) | 1/18 (5.6) (0.1, 27.3) |
| Uterine fibroid type |  |  |
| Subserosal | 16/17 (94.1) (71.3, 99.9) | 0/16 (0) (0.0, 20.6) |
| Intramural | 37/41 (90.2) (76.9, 97.3) | 1/37 (2.7) (0.1, 14.2) |
| Submucosal | 3/4 (75.0) (19.4, 99.4) | 0/2 (0) (0.0, 84.2) |
| Cervical | – | 0/1 (0) (0.0, 97.5) |

BMI = body mass index; CI = confidence interval; PBAC = Pictorial Blood Loss Assessment Chart.

^a^Post hoc analysis.

## Supplemental Table 4

Proportion of patients with a maximum NRS score ≤1 in the 28 days prior to end of treatment in subgroups stratified by baseline factors (full analysis set)

| **n/N (%) (95% CI)** | **Linzagolix (n=48)** | **Placebo (n=41)** |
| --- | --- | --- |
| Age (years) |  |  |
| <40 | 8/14 (57.1) (28.9, 82.3) | 1/6 (16.7) (0.4, 64.1) |
| ≥40 | 26/34 (76.5) (58.8, 89.3) | 3/35 (8.6) (1.8, 23.1) |
| BMI (kg/m^2^) |  |  |
| <25 | 24/36 (66.7) (49.0, 81.4) | 3/23 (13.0) (2.8, 33.6) |
| ≥25 | 10/12 (83.3) (51.6, 97.9) | 1/18 (5.6) (0.1, 27.3) |
| Maximum NRS score |  |  |
| ≥4 to <7 | 15/22 (68.2) (45.1, 86.1) | 2/25 (8.0) (1.0, 26.0) |
| ≥7 | 19/26 (73.1) (52.2, 88.4) | 2/16 (12.5) (1.6, 38.3) |
| Comorbid endometriosis^a^ |  |  |
| No | 32/42 (76.2) (60.5, 87.9) | 4/41 (9.8) (2.7, 23.1) |
| Yes | 2/6 (33.3) (4.3, 77.7) | – |
| Birth experience |  |  |
| No | 16/22 (72.7) (49.8, 89.3) | 2/20 (10.0) (1.2, 31.7) |
| Yes | 18/26 (69.2) (48.2, 85.7) | 2/21 (9.5) (1.2, 30.4) |
| Uterine volume (cm^3^) |  |  |
| ≤170 | 18/27 (66.7) (46.0, 83.5) | 2/23 (8.7) (1.1, 28.0) |
| >170 | 16/21 (76.2) (52.8, 91.8) | 2/18 (11.1) (1.4, 34.7) |
| Uterine fibroid type |  |  |
| Subserosal | 13/17 (76.5) (50.1, 93.2) | 0/16 (0) (0.0, 20.6) |
| Intramural | 30/42 (71.4) (55.4, 84.3) | 4/38 (10.5) (2.9, 24.8) |
| Submucosal | 2/4 (50.0) (6.8, 93.2) | 0/2 (0) (0.0, 84.2) |
| Cervical | – | 0/1 (0) (0.0, 97.5) |

BMI = body mass index; CI = confidence interval; NRS = numeric rating scale.

^a^Post hoc analysis.

## Supplemental Table 5

Secondary efficacy endpoints related to anemia (full analysis set)

|  | **Linzagolix (n=48)** | **Placebo (n=41)** |
| --- | --- | --- |
| Patients with Hb ≥12 g/dL at week 12, n/N (%)^a^ | 9/10 (90.0) | 3/7 (42.9) |
| Blood hematocrit (%), mean (SD) |  |  |
| Baseline | 37.8 (3.4) | 38.7 (3.2) |
| Week 12 | 40.6 (2.8) | 38.7 (4.0) |
| Serum iron (µg/dL), mean (SD) |  |  |
| Baseline | 56.2 (33.2) | 72.5 (48.0) |
| Week 12 | 90.7 (37.4) | 83.6 (46.5) |
| Serum ferritin (ng/mL), mean (SD) |  |  |
| Baseline | 25.0 (19.1) | 33.7 (33.3) |
| Week 12 | 41.2 (27.1) | 27.8 (26.5) |

Hb = hemoglobin; SD = standard deviation.

^a^Analysis included patients with Hb <12 g/dL at baseline and receiving oral iron supplementation.

**Supplemental Table 6**

Summary of pharmacodynamic outcomes (full analysis set)

|  | **Linzagolix (n=48)** | **Placebo (n=41)** |
| --- | --- | --- |
| Plasma estradiol (pg/mL), mean (SD) |  |  |
| Baseline^a^ | 57.6 (42.7) | 45.0 (34.1) |
| Week 4 | 14.7 (66.3) | 97.1 (106.1) |
| Week 8 | 5.8 (8.4) | 146.6 (166.1) |
| Week 12 | 6.4 (8.9) | 131.2 (101.0) |
| Week 16^b^ | 167.7 (142.7) | 130.8 (121.6) |
| Serum FSH (mIU/mL), mean (SD) |  |  |
| Baseline^a^ | 7.9 (3.6) | 10.7 (8.7) |
| Week 4 | 3.9 (2.0) | 10.5 (12.0) |
| Week 8 | 4.0 (2.1) | 8.6 (8.1) |
| Week 12 | 3.8 (2.0) | 8.0 (6.8) |
| Week 16^b^ | 4.4 (3.0) | 10.0 (9.4) |
| Serum LH (mIU/mL), mean (SD) |  |  |
| Baseline^a^ | 3.4 (1.4) | 4.1 (3.0) |
| Week 4 | 1.0 (1.2) | 5.1 (5.2) |
| Week 8 | 1.2 (1.1) | 5.9 (6.1) |
| Week 12 | 1.4 (1.3) | 6.3 (8.7) |
| Week 16^b^ | 3.6 (1.7) | 7.3 (7.7) |
| Serum progesterone (ng/mL), mean (SD) |  |  |
| Baseline^a^ | 0.2 (0.1) | 0.3 (0.3) |
| Week 4 | 0.1 (0.1) | 1.5 (3.3) |
| Week 8 | 0.1 (0.1) | 3.0 (5.1) |
| Week 12 | 0.1 (0.0) | 3.2 (5.0) |
| Week 16^b^ | 7.6 (9.5) | 3.6 (4.7) |

FSH = follicle-stimulating hormone; LH = luteinizing hormone; SD = standard deviation.

^a^Baseline pharmacodynamic tests were performed at the start of the treatment period, which occurred on day 1–5 of menstruation.

^b^Week 16 represents week 4 of the follow-up period.

## Supplemental Figure 1

Study design

QID = once daily; R = randomization.

^a^The screening period was defined as the time between the screening visit and the next visit during day 1–5 of the first menstruation after screening (visit 2); the run-in period was defined as the time between visit 2 and the next visit during day 1–5 of the second menstruation after screening (visit 3).

^b^For patients who discontinued study treatment before week 12, the follow-up period was 4 weeks after the last dose.

^c^Weeks after the start of the treatment period.

## Supplemental Figure 2

Patient disposition

FAS = full analysis set; SS = safety set.

^a^The run-in period was defined as the time between day 1–5 of the first menstruation after screening (visit 2) and day 1–5 of the second menstruation after screening (visit 3).

## Supplemental Figure 3

Kaplan–Meier plot of time to a total PBAC score <10 (full analysis set)

The total PBAC score was calculated every 42 days (i.e., day 1–42, 2–43, 3–44, etc.), and the event onset date was defined as the first day of the 42-day period in which a total PBAC score <10 was achieved and maintained through to the end of study treatment. Circles represent censored patients; time to a total PBAC score <10 was significantly shorter in the linzagolix group versus placebo (*P*<0.001, based on log-rank tests). PBAC = Pictorial Blood Loss Assessment Chart.

## Supplemental Figure 4

Kaplan–Meier plot of time to a maximum NRS score ≤1 (full analysis set; post hoc analysis)

The maximum NRS score was evaluated every 28 days (i.e., day 1–28, 2–29, 3–30, etc.), and the event onset date was defined as the first day of the 28-day period in which a maximum NRS score ≤1 was achieved and maintained through to the end of study treatment. Circles represent censored patients; time to a maximum NRS score ≤1 was significantly shorter in the linzagolix group versus placebo (*P*<0.001, based on log-rank tests). NRS = numeric rating scale.

## Supplemental Figure 5

Mean plasma estradiol levels over time (full analysis set)

Week 0–12 represents the treatment period; week 12–16 represents the follow-up period. Error bars represent standard deviation.
